# Epidemiology, Hot Spots, and Sociodemographic Risk Factors of Alcohol Consumption in Indian Men and Women: Analysis of National Family Health Survey-4 (2015-16), a Nationally Representative Cross-Sectional Study

**DOI:** 10.3389/fpubh.2021.617311

**Published:** 2021-08-27

**Authors:** Karuppusamy Balasubramani, Winnie Paulson, Savitha Chellappan, Ramakrishnan Ramachandran, Sujit Kumar Behera, Praveen Balabaskaran Nina

**Affiliations:** ^1^Department of Geography, Central University of Tamil Nadu, Thiruvarur, India; ^2^Department of Epidemiology and Public Health, Central University of Tamil Nadu, Thiruvarur, India; ^3^National Institute of Traditional Medicine (ICMR), Belagavi, India

**Keywords:** alcohol consumption in India, spatial statistics, Getis-Ord Gi^*^, alcohol hot spots in India, NFHS-4

## Abstract

**Objectives:** To map the alcohol hot spots and understand the Sociodemographic Indices (SDI) affecting alcohol consumption in Indian men and women.

**Methods:** Data from National Family Health Survey-4 carried out from 2015 to 2016 with a sample size of 103,411 men and 699,686 women were used for Geographic Information System mapping, and hot spot identification by spatial statistics (Getis-Ord Gi^*^). Bivariate analyses and multiple logistic regressions were used to analyze SDI.

**Results:** India has three major alcohol hot spots: (1) North-East (NE) states, (2) Eastern Peninsular states formed by Chhattisgarh, Odisha, Jharkhand, and Telangana, and (3) Southern states of Tamil Nadu and Kerala. Hot spot analysis strongly correlated with region-wise analysis of SDI. Respondents who consumed tobacco have higher odds (men adjusted odds ratio [aOR]: 5.42; women aOR: 4.30) of consuming alcohol. Except for religion and social category, other socioeconomic factors have a low to moderate effect on alcohol consumption.

**Conclusions:** Hot spots and high-risk districts of alcohol consumption identified in this study can guide public health policies for targeted intervention. Alcohol use is at the discretion of individual states and union territories, and stringent anti-alcohol policies strictly enforced across India are the keys to control alcohol use.

## Background

As per WHO, worldwide, an estimated annual three million deaths and 5.1% of Disability-Adjusted Life Years (DALYs) of the world are attributed to alcohol consumption (1). Alcohol consumption is a leading cause of global disease burden and health loss, and in 2016, it was ranked seventh in risk factors linked to deaths and DALYs (2). Alcohol use has been linked to 60 acute and chronic disease conditions, especially cancers, liver cirrhosis, and cardiovascular diseases such as myocardial infarction, ischemic stroke, elevated blood pressure, coronary heart disease, and hemorrhagic stroke (3–8). Alcohol is also indirectly related to an increase in road accidents (9). The global alcohol use and burden study show that health loss is associated with all levels of consumption (2), challenging few studies that suggest a low level of alcohol consumption may be protective against certain disease outcomes (10–12).

In India, alcohol consumption is widespread across all the states and the union territories (UT), and an estimated 160 million consume alcohol (13). According to National Family Health Survey-4 (NFHS-4), 29.2 men and 1.2% women consume alcohol (14). Alcohol use in India is estimated to cause an annual average loss of 1.45% of the gross domestic product (GDP) of the economy (15). India does not have a solid national policy on alcohol consumption, and its use is regulated at the level of individual states and UT. Other than a geospatial study of alcohol use based on 1998's Special Fertility and Mortality Survey (16), very little information is available on the statistically significant hot spot clusters of alcohol consumption at the district level in India. Even though earlier studies have reported the socioeconomic determinants of co-use of alcohol and tobacco, and substance use among North-East (NE) men (17, 18), the sociodemographic risk factors of alcohol use in Indian men (or women) have not been studied. The data from NFHS-4 were used for Geographic Information System (GIS) and spatial statistics to map the major alcohol hot spots in India. In addition, the high-risk states and districts in the hot spots have also been identified for targeted intervention. Furthermore, the Sociodemographic Indices (SDI) have been analyzed separately in men and women to identify the risk factors that influence alcohol consumption.

## Methods

### Data Source

The datasets of NFHS-4 carried out by the International Institute of Population Sciences from January 20, 2015, to December 4, 2016, were used for analysis (14). The data from NFHS-4 are representative of each of 640 districts from 29 states and 7 UT of India for many key health indicators, which also include alcohol consumption. A multistage cluster sampling was used for data collection. The 2011 census served as the sampling frame for the selection of villages and households in each district. From the randomly selected households, all the women of ages 15–49 years were asked to participate in the survey. Men of ages 15–54 years were invited to participate from a random subsample of 15% of these households. The overall sample size of men was 103,411, women was 699,686, and the age-group was 15–49 years. The women respondents were considerably higher because the main focus of NFHS-4 was on maternal and child health. The NFHS-4 data were collected using Computer Assisted Personal Interviewing (CAPI) on mini-notebook computers by trained interviewers. Permission to use the NFHS-4 data was obtained from the Demographic and Health Survey program (DHS 2020). The administrative boundary and population data of 640 districts of India were obtained from the census of India to prepare a spatial database in GIS (19). The NFHS-4 survey was funded by United States Agency for international development. United Nation's Children Fund also assisted the project with supplementary funding support.

### Outcome Variable

The outcome of interest in this study is the use of alcohol by the respondents. This was based on questions: 1. Do you drink alcohol? (Response was categorized as “yes” or “no”) and 2. What type of alcohol do you usually drink? The options given were tadi madi, country liquor, beer, wine, hard liquor, and others.

### Explanatory Variables

The SDI used for this analysis are the place of residence (rural or urban), religion (Hindu, Muslim, Christian, and others), age-group (15–19, 20–34, and 35–49 years), states are grouped into region (North, Central, East, North-East, West, and South), social group (scheduled castes, scheduled tribes (STs), other backward classes, and others), wealth quintile (poorest, poorer, middle, richer, and richest), marital status (never married, currently married, and others), educational level (no education, primary, secondary, and higher), and occupation (not working, professional/managerial, clerical/sales/service, agriculture, and skilled/unskilled manual). The independent variables were selected after a prior literature search (20).

### Statistical Analysis

Descriptive and bivariate statistical analyses were carried out to estimate the prevalence of alcohol consumption under the different socioeconomic and demographic variables. The missing values (<1%) were not included in the analysis. The variables for the multivariate regression analysis were based on the purposeful selection of variables (21). At first, univariate analysis was carried out, and any variable significantly associated at *p* < 0.25 with alcohol use was qualified as a potential candidate variable for the multivariate model. In the iterative process of variable selection, covariates were removed from the model if they were non-significant at *p* < 0.10 and not a confounder (a change in the odds ratio of remaining variables by 15% or more). In the final step, any variable (insignificant in univariate analysis) not selected for the original multivariate model was added back one at a time, with significant covariates and confounders retained earlier. Variables that are significant at the 0.10 level were analyzed in the model. Survey weights computed in the NFHS were used in the analysis. STATA version 16.0 (StataCorp LLC) was used for analyses, and the results are considered significant at alpha level 5%, and 95% confidence intervals are reported for the estimates.

### Spatial Analysis

The district and state level prevalence of alcohol use of men and women was linked to administrative boundaries of India using ArcGIS software. This linked spatial database was used to prepare thematic maps to represent the spatial distribution of alcohol use by men and women in India. To assess how well the district-wise distribution of alcohol consumption correlates with nearby districts across the country, we tried a spatial autocorrelation approach. This spatial statistic approach is generally used to describe the presence of systematic spatial variations in a region, and to identify high-risk clusters within the region for focused action (22). Join-count statistic, Moran's I, Getis-Ord Gi^*^, Geary's C, spline-based models, simultaneous autoregressive models, and Gaussian Markov random field are among the many spatial autocorrelation approaches used for describing how a variable is autocorrelated through space (23). Among these, Getis-Ord Gi^*^ is a highly preferred approach to measure the level of spatial interdependence between the variables, and the nature and strength of that interdependence (24). It is one of the widely used GIS-based tool to find hot spots of phenomena under investigation (25). Thus, to understand the hot spots of alcohol consumption in India, we used Getis-Ord Gi^*^ spatial autocorrelation tool in this study. The tool calculates *z*-scores (standard deviations) and *p*-values (probability) for each district within the context of neighboring districts (26). In the normal distribution curve, very high (positive) or very low (negative) *z*-scores with very small *p*-values populate in the tails. These positive and negative *z*-scores are termed as hot and cold spots, respectively. In this analysis, the inverse distance squared function was used where closer districts are weighed more heavily than the districts far away. The spatial autocorrelation was executed separately for men and women to identify their hot spots in alcohol consumption. To highlight vulnerable districts/states in terms of alcohol prevalence, a scatter-plot matrix representing men density and alcohol consumption was prepared in GIS. To understand the distribution of different types of alcohol use, a state-wise stacked bar diagram was prepared for both men and women.

## Results

### Spatial Distribution of Alcohol Consumption

**Figure 1:**
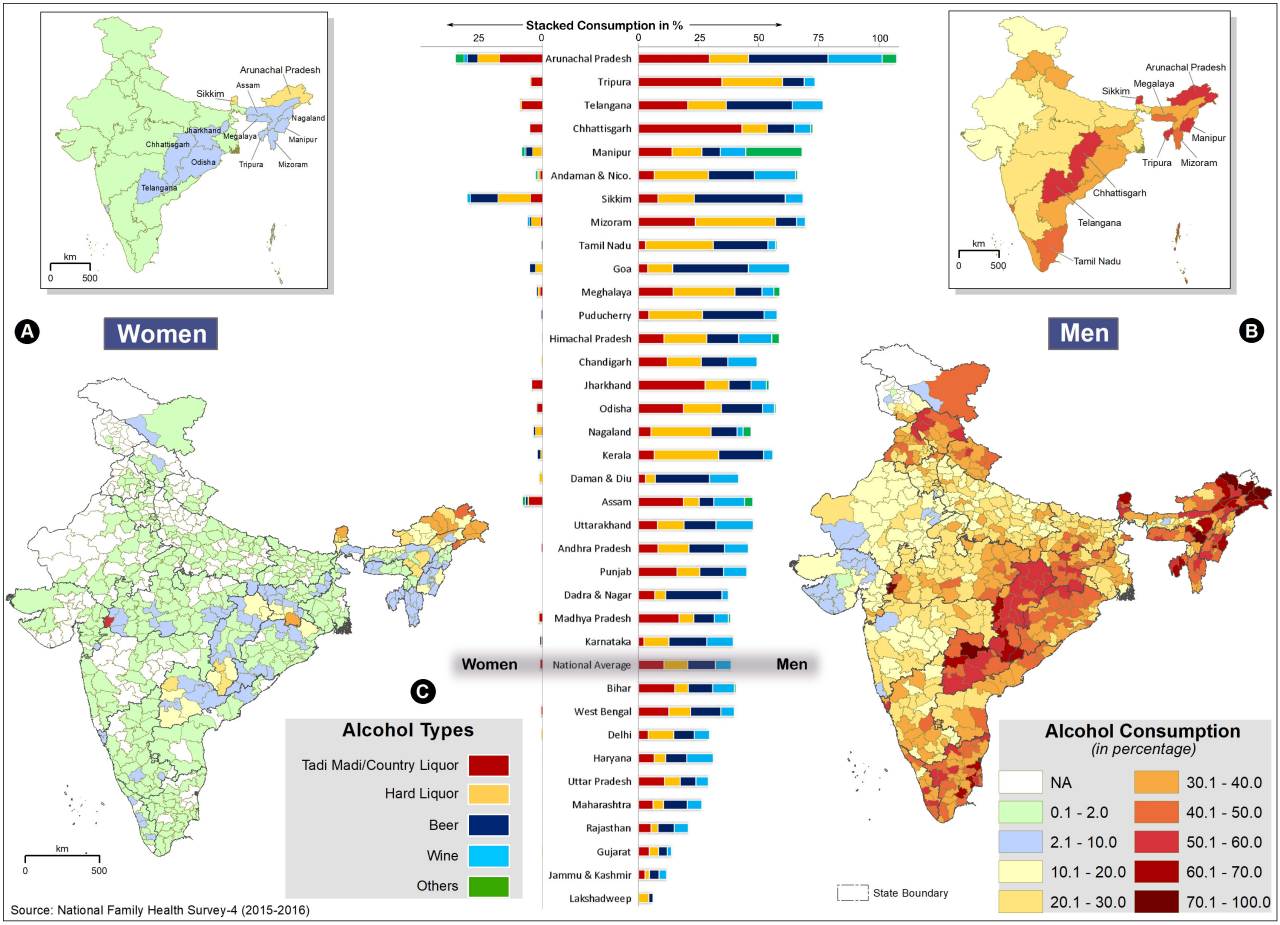
State and district-wise comparison of alcohol consumption in men and women, National Family Health Survey (NFHS)-4. The inset maps (top) show the state-wise average, and the bottom maps show the district-wise alcohol distribution for women **(A)** and men **(B)**. The darker red shades denote higher consumption. The state boundaries are overlaid to show inter-intra state distributions. The state-wise comparison of different types of alcohol consumption is shown for men (right bars) and women (left bars) **(C)**. The colors in the bars indicate relative consumption (%) of different alcohol types.

The state- and district-wise comparison of alcohol consumption prevalence in men and women recorded by NFHS-4 is shown in Figure 1. On an average, only 1.2% of women in India consume alcohol, whereas the national average for men is 29.2%. Alcohol consumption by both men and women is high in NE and Eastern states of India. In addition, alcohol consumption by men is high in South and North India. In men, high-consuming alcohol states are Arunachal Pradesh, Tripura, Telangana, Chhattisgarh, Manipur, Sikkim, Mizoram, and Tamil Nadu where >45% of men consume alcohol. Alcohol prevalence among women is high in all NE states and Telangana.

The district-wise distribution of alcohol consumption in India shows that excluding a few isolated districts of Gujarat, Rajasthan, and Jammu and Kashmir, all the districts of India have alcohol prevalence in excess of 10% in men (Figure 1B, Supplementary Table 2). Alcohol use is very high (>50%) in NE India and Eastern peninsular states, especially Telangana and Chhattisgarh. In some districts of Arunachal Pradesh (Anjaw and Upper Siang), the prevalence is 100%. Except for NE, Telangana, and border districts in Jharkhand, Chhattisgarh, and Odisha, the alcohol consumption among women in all the other districts is quite low (<2%). However, many districts in Arunachal Pradesh exceed 30%, and the highest prevalence of alcohol consumption in women (53.2%) was observed in Alirajpur district of Madhya Pradesh (Figure 1A, Supplementary Table 2).

In India, both men and women, consume different types of alcoholic beverages such as tadi madi, country liquor, hard liquor (spirit), beer, wine, or other alcoholic beverages, and the state-wise distribution is given in Figure 1C. In all the states, more than one type of alcohol is consumed, and overall, there is no single dominant alcoholic beverage in India. However, hard liquor with high alcohol content is highly consumed in Mizoram, Tamil Nadu, Meghalaya, Nagaland, and Kerala. The proportion of tadi madi/country liquor is higher in the tribal- dominated states of Tripura, Chhattisgarh, Jharkhand, Assam, Odisha, and Madhya Pradesh. Compared to men, women in these states mostly prefer tadi madi/country liquor. Beer and wine constitute the largest proportion of alcohol consumed in the small states (Sikkim and Goa) and the UT (Andaman and Nicobar, Daman and Diu, Dadra and Nagar, and Puducherry).

### Hot Spots of Alcohol Consumption

**Figure 2:**
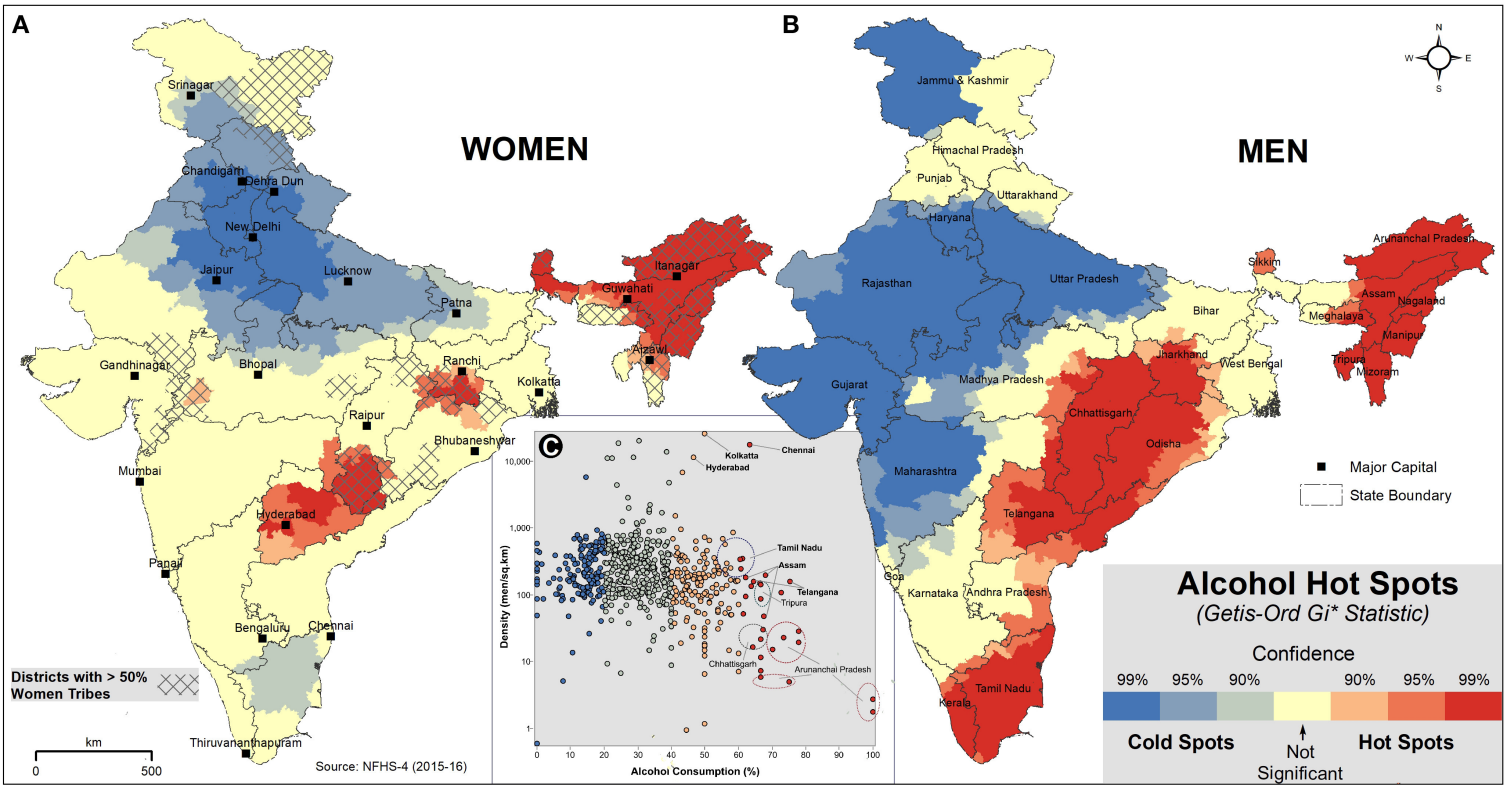
Hot spot analysis of alcohol consumption in India, NFHS-4. The hot (red) and cold (blue) spots of alcohol consumption with three confidence levels (99, 95, and 90%) are shown for women **(A)** and men **(B)**. The light yellow color shows the spatially not-significant districts of alcohol consumption. The overlaid hatches **(A)** indicate that more than 50% of women are ST in that district. Scatter plot **(C)** shows the relationship between alcohol consumption and density. The dots represent district values of alcohol consumption of men (%) and men density per sq.km.

We employed Getis-Ord Gi^*^ statistic to identify statistically significant spatial clusters of alcohol prevalence among men and women. To form a statistically significant hotspot, the district with high prevalence should be surrounded by districts with high prevalence. Based on *z*-scores and *p*-values, hot spots of alcohol consumption for men and women are shown with 99, 95, and 90% confidence intervals (Figures 2A,B, Supplementary Table 1). There are three significant alcohol hot spots in India. NE states form the first and prominent hot spot of alcohol consumption in India for both men and women. Due to a lack of data from the neighboring districts, some of the border districts in the NE hot spot are excluded from the results. However, individual consumption rates of those districts are high, and hence we consider the entire NE region as a hot spot. The second hot spot is formed primarily by the districts of Chhattisgarh, Odisha, Jharkhand, and Telangana states of the Eastern peninsular region. This hot spot is more pronounced in men, and also extends to the western districts of Madhya Pradesh and North-Eastern districts of Andhra Pradesh. In women, the second hot spot is split into two, the districts in the borders of Jharkhand, Chhattisgarh, and Odisha form one cluster, and the second cluster is formed by Telangana (excluding the Northern districts) and the Southern districts of Jharkhand. Most of the districts in NE and Eastern peninsular hot spots (Jharkhand, Chhattisgarh, and Odisha) have a high proportion of STs (>75%). The bifurcation of the alcohol hot spot of women in the Eastern peninsular region coincides with the distribution of the ST women population (Figure 2A).

The third hot spot is formed by the districts of Tamil Nadu and South Kerala, and it is seen only in men. Tamil Nadu is a cold spot for women. The cold spots cover the majority of the districts in Western and Northern India, excluding the states of Punjab, Himachal Pradesh, and Uttarkhand, where alcohol consumption is moderately high.

Even though the consumption of alcohol is very high in NE, population density in this region, especially in Arunachal Pradesh and Tripura, is very low compared to the rest of India. In order to identify vulnerable districts/states in terms of population density and high alcohol prevalence, scatter plot analysis was carried with the district-level consumption in men (%) and men density (per sq.km) (Figure 2C). The analysis shows that among the three hot spots, the districts of Tamil Nadu, Telangana, and Assam are highly vulnerable. In the high-risk metro districts of Chennai (Tamil Nadu), Hyderabad (Telangana), and Kolkata (West Bengal), the men density exceeds 10,000 per sq.km, and the alcohol consumption rate is more than 40%.

### Sociodemographic Indices Affecting Alcohol Consumption in Indian Men and Women

The sociodemographic profile of the respondents aged 15–49 years is given in Table 1. The prevalence of alcohol consumption among men and women aged 15–49 years by different SDI and aOR is given in Tables 2, 3, respectively.

**Table 1:** Frequency and percentage distribution of men and women responders (15–49 years) by sociodemographic characteristics, National Family Health Survey (NFHS)−4 (2015-2016).

**Socioeconomic characteristics**	**Men**		**Women**	
	***n***	**%**	***n***	**%**
**Place of residence**				
Rural	63,864	61.8	457,461	65.4
Urban	39,546	38.2	242,225	34.6
**Age**				
15–24	35,363	34.2	244,517	34.9
25–39	44,545	43.1	302,701	43.3
40–49	23,501	22.7	152,466	21.8
**Region**				
North	14,850	14.4	95,098	13.6
Central	22,467	21.7	165,474	23.6
East	19,279	18.6	154,698	22.1
North- East	3,398	3.3	24,615	3.5
West	19,146	18.5	100,535	14.4
South	24,271	23.5	159,266	22.8
**Educational level**				
No education	12,234	11.8	192,135	27.5
Primary	12,400	12.0	87233	12.5
Secondary	60,350	58.4	331,037	47.3
Higher	18,428	17.8	89,281	12.8
**Religion**				
Hindu	84,211	81.4	563,739	80.6
Muslim	13,793	13.3	96,461	13.8
Christian	2,277	2.2	16,620	2.4
Others	3,130	3	22,866	3.3
**Social category**				
Others	24,047	24.3	157,774	23.6
Schedule caste	20,498	20.8	142,619	21.3
Schedule tribe	9,132	9.2	64,144	9.6
Other backward classes	45,110	45.7	303,837	45.5
**Wealth index**				
Poorest	15,205	14.7	124,054	17.7
Poorer	19,402	18.8	136,900	19.6
Middle	22,047	21.3	143,814	20.6
Richer	22,930	22.2	147,978	21.1
Richest	23,827	23	146,939	21
**Marital status**				
Never married	39,631	38.3	157,136	22.5
Married	62,499	60.4	513,271	73.4
Others	1,280	1.2	29,279	4.2
**Occupation**				
Not working	23,861	23.1	84,228	70.6
Professional/Managerial	6,636	6.4	3,516	2.9
Clerical/Sales/Services	19,023	18.4	6,405	5.4
Agricultural	26,753	25.9	17,717	14.8
Skilled/Unskilled manual	26,949	26.1	7,592	6.3
**Alcohol consumption**				
Yes	30,166	29.2	8,638	1.2
No	73,244	70.8	691,048	98.8

**Table 2:** Prevalence of alcohol use among survey participants aged 15–49 years by socioeconomic characteristics, India, NFHS-4.

**Socioeconomic characteristics**	**Alcohol men**			**Alcohol women**		
	**Yes (%)**	**No (%)**	**Total**	**Yes (%)**	**No (%)**	**Total**
**Place of residence**						
Rural	18,827 (29.5)	45,037 (70.5)	63,864	6,920 (1.5)	450,541 (98.5)	457,461
Urban	11,339 (28.7)	28,207 (71.3)	39,546	1,717 (0.7)	240,507 (99.3)	242,224
**Age**						
15–24	5,686 (16.1)	29,677 (83.9)	35,363	1,516 (0.6)	243,001 (99.4)	244,517
25–39	15,865 (35.6)	28,680 (64.4)	44,545	3,992 (1.3)	298,709 (98.7)	302,701
40–49	8,614 (36.7)	14,886 (63.3)	23,500	3,128 (2.1)	149,338 (97.9)	152,466
**Region**						
North	3,433 (23.1)	11,416 (76.9)	14,849	171 (0.2)	94,927 (99.8)	95,098
Central	6,149 (27.4)	16,318 (72.6)	22,467	1,566 (1.0)	163,784 (99)	165,350
East	6,117 (31.7)	13,162 (68.3)	19,279	1,911 (1.2)	152,786 (98.8)	154,697
North- East	1,376 (40.5)	2,021(59.5)	3,397	1,561(6.9)	22,914 (93.1)	24,615
West	3,246 (17.0)	15,900 (83)	19,146	277 (0.3)	100,258 (99.7)	100,535
South	9,842 (40.6)	14,428 (59.4)	24,270	2,887 (1.8)	156,378 (98.2)	159,265
**Educational level**						
No education	4,876 (39.9)	7,358 (60.1)	12,234	5,074 (2.6)	187,061 (97.4)	192,135
Primary	4,537 (36.6)	7,862 (63.4)	12,399	970 (1.1)	86,262 (98.9)	87,232
Secondary	16,337 (27.1)	44,012 (72.9)	60,349	1,977 (0.6)	329,060 (99.4)	33,1037
Higher	4,415 (24.0)	14,012 (76)	18,427	615 (0.7)	88,665 (99.3)	89,280
**Religion**						
Hindu	26,585 (31.6)	57,625 (68.4)	84,210	7,323 (1.3)	556,415 (98.7)	563,738
Muslim	1,561 (11.3)	12,232 (88.7)	13,793	137 (0.1)	96,324 (99.9)	96,461
Christian	973 (42.8)	1,303 (57.2)	2,276	668 (4.0)	152,83 (96)	15,951
Others	1,046 (33.4)	2,084 (66.6)	3,130	508 (2.2)	22,358 (97.8)	22,866
**Social category**						
Others	5,188 (21.6)	18,859 (78.4)	24,047	754 (0.5)	157,019 (99.5)	157,773
Schedule caste	7,442 (36.3)	13,056 (63.7)	20,498	1,251 (0.9)	141,367 (99.1)	142,618
Schedule tribe	3,775 (41.3)	5,357 (58.7)	9,132	4,145 (6.5)	59,999 (93.5)	64,144
Other backward classes	12,872 (28.5)	32,238 (71.5)	45,110	2,229 (0.7)	301,608 (99.3)	303,837
**Wealth index**						
Poorest	5,387 (35.4)	9,818 (64.6)	15205	3,596 (2.9)	120,458 (97.1)	124,054
Poorer	5,796 (29.9)	13,606 (70.1)	19402	1,931 (1.4)	134,969 (98.6)	136,900
Middle	6,623 (30.0)	15,423 (70)	22,046	1,367 (1.0)	142,446 (99)	143,814
Richer	6,376 (27.8)	16,553 (72.2)	22,929	829 (0.6)	147,149 (99.4)	147,978
Richest	5,982 (25.1)	17,844 (74.9)	23,826	911 (0.6)	146,028 (99.4)	146,939
**Marital status**						
Never married	7,406 (18.7)	32,225 (81.3)	39,631	1,035 (0.7)	156,101 (99.3)	157,136
Married	22,242 (35.6)	40,257 (64.4)	62,499	6,765 (1.3)	506,506 (98.7)	513,271
Others	517 (40.4)	763 (59.6)	1,280	837 (2.9)	28,441 (97.1)	29,278
**Occupation**						
Not working	3,322 (13.9)	20,539 (86.1)	23,861	607 (0.7)	83,821 (99.3)	84,428
Professional/Managerial	1,801 (27.1)	4,835 (72.9)	6,636	43 (1.2)	3,473 (98.8)	3,516
Clerical/Sales/Services	6,236 (32.8)	12,787 (67.2)	19,023	123 (1.9)	6,282 (98.1)	6,405
Agricultural	8,607 (32.2)	18,146 (67.8)	26,753	535 (3.0)	17,182 (97)	17,717
Skilled/Unskilled manual	10,148 (37.7)	16,801 (62.3)	26,949	185 (2.4)	7,407 (97.6)	7,592
**Tobacco consumption**						
Yes	21,291 (46.3)	24,718 (53.7)	46,009	3,394 (7.1)	44,156 (92.9)	47,550
No	8,875 (15.5)	48,526 (84.5)	57,401	5,244 (0.8)	64,6891 (99.2)	652,135

**Table 3:** Socioeconomic and demographic characteristics of alcohol consumption, India, NFHS-4.

**Socioeconomic characteristics**	**Alcohol men**			**Alcohol women**		
	**Unadjusted odds ratio**	**Adjusted odds ratio (aOR)**	**95% CI^a^**	**Unadjusted odds ratio**	**Adjusted odds ratio (aOR)**	**95% CI**
**Tobacco consumption**						
No	Reference (1.0)	Reference (1.0)	Reference (1.0)	Reference (1.0)	Reference (1.0)	Reference (1.0)
Yes	4.74	5.42^**^	5.05–5.81	9.48	4.30^**^	3.48–5.32
**Age**						
15–24	Reference (1.0)	Reference (1.0)	Reference (1.0)	Reference (1.0)	Reference (1.0)	Reference (1.0)
25–39	2.89	1.70^**^	1.57–1.85	2.14	1.18	0.93–1.49
40–49	3.02	1.56^**^	1.42–1.71	3.36	1.47^**^	1.15–1.88
**Region**						
South	Reference (1.0)	Reference (1.0)	Reference (1.0)	Reference (1.0)	Reference (1.0)	Reference (1.0)
Central	0.56	0.30^**^	0.28–0.33	0.56	0.28^**^	0.21–0.37
East	0.69	0.41^**^	0.37–0.46	0.68	0.29^**^	0.21–0.40
West	0.30	0.16^**^	0.14–0.19	0.15	0.08^**^	0.05–0.14
North	0.45	0.31^**^	0.28–0.34	0.10	0.09^**^	0.03–0.23
North-East	1.01	0.61^**^	0.53–0.70	4.02	2.07^**^	1.45–2.95
**Educational level^c^**						
No education	Reference (1.0)			Reference (1.0)	Reference (1.0)	Reference (1.0)
Primary	0.88			0.41	0.53^**^	0.42–0.67
Secondary	0.56			0.22	0.44^**^	0.36–0.54
Higher	0.48			0.26	0.57^**^	0.37–0.88
**Religion^b^**						
Hindu	Reference (1.0)	Reference (1.0)	Reference (1.0)	Reference (1.0)	Reference (1.0)	Reference (1.0)
Muslim	0.28	0.24^**^	0.21–0.28	0.11	0.17^**^	0.07–0.42
Christian	1.61	1.09	0.93–1.27	3.18	0.71	0.45–1.12
Others	1.09	2.03^**^	1.71–2.42	1.73	2.74^**^	2.05–3.67
**Social category**						
Schedule caste	Reference (1.0)	Reference (1.0)	Reference (1.0)	Reference (1.0)	Reference (1.0)	Reference (1.0)
Schedule tribe	1.24	1.37^**^	1.24–1.51	7.81	5.83^**^	4.40–7.73
OBC	0.70	0.82	0.76–0.88	0.84	0.97	0.73–1.30
Others	0.48	0.72	0.66–0.80	0.54	1.47^*^	1.01–2.13
**Wealth index**						
Poorest	Reference (1.0)	Reference (1.0)	Reference (1.0)	Reference (1.0)	Reference (1.0)	Reference (1.0)
Poorer	0.78	0.85^**^	0.79–0.91	0.48	0.53^**^	0.42–0.67
Middle	0.78	0.90^**^	0.83–0.97	0.32	0.45^**^	0.35–0.60
Richer	0.70	0.87^**^	0.80–0.96	0.19	0.48^**^	0.34–0.68
Richest	0.61	1.00	0.90–1.11	0.21	0.56^**^	0.46–0.78
**Marital status**						
Never married	Reference (1.0)	Reference (1.0)	Reference (1.0)	Reference (1.0)		
Married	2.40	1.09^*^	1.00–1.18	2.01		
Others	2.98	1.23	0.98–1.55	4.44		
**Occupation**						
Not working	Reference (1.0)	Reference (1.0)	Reference (1.0)	Reference (1.0)	Reference (1.0)	Reference (1.0)
Professional/Managerial	2.34	1.50^**^	1.31–1.72	1.73	1.33	0.72–2.47
Clerical/Sales/Services	3.04	1.90^**^	1.70–2.11	2.71	1.62^*^	1.10–2.38
Agricultural	2.95	1.43^**^	1.31–1.57	4.30	1.85^**^	1.51–2.26
Skilled/Unskilled manual	3.75	2.06^**^	1.88–2.26	3.46	1.84^**^	1.48–2.30
**Place of residence^c^**						
Rural	Reference (1.0)	Reference (1.0)	Reference (1.0)	Reference (1.0)		
Urban	0.96	1.18^**^	1.09–1.28	0.46		

a *CI, Confidence Interval*.

b *Religion confounded the association between caste and alcohol use in men*.

c *Education for men; residence and marital status for women were not included in the final model*.

** *p < 0.01*,

* *p < 0.05*.

The outcome variable was alcohol consumption (Yes = 1, No = 0). Ten covariates selected through literature are listed in Table 1, and all the covariates were treated as equally important. The univariable regression analysis identified nine covariates in men (except residence-urban/rural) and all 10 covariates in women as potential candidate variables for the multivariable model at the 0.25 alpha level. During the iterative process of variable selection for the multivariable model, the variables “education” in men and “residence” and “marital status” in women were eliminated one at a time because they were not significant in the multivariable model at the alpha level of 0.10, and when taken out, did not change the odds ratio of any of the remaining variables by more than 15%. The variable “religion” in men was also not significant at the 0.10 alpha level but changed the odds ratio for the caste (other backward classes and other general castes) covariate by more than 15% when taken out; therefore, it was retained in the model as a confounder. The variable, residence, in men set aside initially (univariable analysis) due to lack of significance at the 0.25 alpha level, made it back in the model when tested with the eight retained covariates as it was significant at the 0.10 alpha level.

Men (aOR: 5.42) and women (aOR: 4.30) who use tobacco have higher odds of alcohol consumption than those who do not consume. South Indian men (aOR: 1) and NE men (aOR: 0.61) and women (aOR: 2.06) consume more alcohol. The place of residence (urban or rural) influences the consumption pattern in men and the urban population (aOR: 1.18) tends to use alcohol more. Alcohol consumption among women increases with age, and it is higher in women older than 40 years (aOR: 1.0, 1.18, and 1.47, respectively, for 15–24, 25–39, and >40 years). Alcohol use is higher in men aged 25–39 years (aOR: 1.70). Never-married men consume less alcohol compared to their other counterparts i.e., married (aOR: 1.09) and others (aOR: 1.23).

Women from the poorest wealth index (aOR: 1.00) and uneducated background (aOR: 1.00) have a high prevalence of alcohol consumption compared to the richest and highly educated. When the occupation was analyzed, not working men (aOR: 1.00) and women (aOR: 1.00) consumed less alcohol than working counterparts.

Among the SDI, religion and social categories had the greatest influence on alcohol use. Compared to other religions, Muslim men (aOR: 0.24) and women (aOR: 0.17) consume less alcohol. Among the different social categories in men, STs (aOR: 1.37) and scheduled castes (aOR: 1.00) have higher alcohol prevalence. Compared to the other castes, ST women (aOR: 5.83) have six times higher odds of consuming alcohol.

## Discussions

Alcohol consumption is widespread across India, and an estimated 160 million consume alcohol (13). In addition to the health burden, alcohol consumption in India has serious social consequences such as domestic violence, lower quality of life, a strained relationship with family members, and psychological and emotional effects on children, which adversely impacts education (27, 28). Compared to NFHS-3 (2005–06), alcohol consumption by men has decreased by just 2.7% in NFHS-4 (2015–16), a negligible decline (14). Our findings over the two surveys indicate that alcohol consumption is similar in many of the states, and it has increased in Tamil Nadu, Himachal Pradesh, Tripura, and Mizoram. The lack of a national policy on alcohol control is the major reason for the high alcohol consumption across India. The state governments are empowered to manufacture, distribute, and regulate the sale and use of alcohol (29). Except for Gujarat, Bihar (ban from 2016), Lakshadweep, Mizoram (ban from 2018), and Manipur (partial ban), alcohol consumption is legal in all other states and UT. The effect of the ban on alcohol consumption in Gujarat (Gujarat prohibition act, 1949) (30) is reflected in the low prevalence of alcohol in the state. In Tamil Nadu, the earlier policy of alcohol ban led to numerous deaths due to methanol poisoning linked to locally brewed illicit liquors. Since 2002, alcohol is sold by the Tamil Nadu government through several thousand retail outlets distributed across the state (31). Intriguingly, the legal and easy availability of alcohol had resulted in a substantial increase in alcohol users, and nearly 50% of the men surveyed in NFHS-4 consume alcohol in Tamil Nadu. Alcohol prohibition in Bihar is in force from April 2016, and the prevalence outcome can only be assessed in NFHS-5. Despite the availability of illicit alcohol in the border areas from neighboring states, and a move toward other drugs for addiction (32), the ban when strictly enforced should benefit Bihar and other states, especially the younger generation with no alcohol exposure, as they will not have legal access. The spike in Mizoram in NFHS-4 could be linked to the lifting of the alcohol ban after 18 years in January 2015, the start of the survey period (33). For many state governments, taxation from alcohol is an important source of revenue (29). For example, Tamil Nadu distributes and sells alcohol through its 5,152 own outlets and adds over 30% revenue (>Rs.30,000 crore/year) to its exchequer (34, 35).

NFHS-4 findings indicate that alcohol consumption is seen predominantly in men (29.2%). Peer pressure, habituation, stress, and tiredness from work are attributed as reasons for alcohol use in Indian men (27). Social and cultural characteristics of the Indian society greatly limit the consumption of alcohol by Indian women (36); only 1.2% women consume alcohol. Prevalence of alcohol was higher in men between 25 and 39 years (35.6%), and as seen in many countries (37), did not decrease with age (40–49 years−36.7%); these findings suggest that men are addicted.

The district-wise distribution and subsequent hot spot analysis show that the NE region forms the first hot spot. Almost all the districts of Chhattisgarh, Odisha, Telangana, and Northeastern Andhra Pradesh constitute the second hot spot, and the third hot spot is made up by all the districts of Tamil Nadu and Southern districts of Kerala. The NE region shares porous international borders with Myanmar and Bangladesh, and the easy availability of cheap tobacco and alcohol in NE smuggled through these countries could be a major reason for the high prevalence (38). Even though the consumption of alcohol is very high in the NE states, the alcohol consumers per sq.km are less (except in Assam). Among the hot spots, based on population density and alcohol consumption, the high-risk districts are in Tamil Nadu, Telangana, and Assam. In addition, the high proportion of hard liquor (contains ~40% alcohol by volume) consumers in Tamil Nadu and Telangana strongly indicate addiction. Not surprisingly, road accidents associated with drunken driving in Tamil Nadu are among the highest in India (39).

Our analysis shows that most of the SDI have a low to moderate association with alcohol use. Religion and social category had a major influence on alcohol consumption. Alcohol use is lower in Muslims (Men−11.3%; Women−0.1%), as Islam prohibits alcohol consumption (40). Alcohol and tobacco co-use exists, since in both men (aOR: 5.42) and women (aOR: 4.30), those who consume tobacco have higher odds of alcohol consumption. Our findings indicate that most of the districts with high consumption of alcohol, especially women alcohol hot spots, coincide with districts with a high proportion of ST population. The multilogistic regression analysis also indicates a strong association between ST population and alcohol consumption by men (aOR: 1.37) and women (aOR: 5.83); 75% of alcohol consuming women are ST. In women, high alcohol consumption is also seen among the poorest, individuals with no education, and in manual/agricultural workers. ST have very low literacy rates, are most economically backward, and a majority of them are manual/agricultural workers (41). Overall, except for region (South—men; NE-men and women), religious faith (Islam), and social category (SC and ST—men; ST—women), the other sociodemographic determinants do not appear to strongly affect alcohol consumption.

As the study is based on a cross-sectional survey, a major limitation is it is not possible to measure the direction of association between the sociodemographic characteristics and the outcome variables. Furthermore, alcohol use was self-reported by the survey participants, hence there is a chance of under- or overreporting. Even though the majority of alcohol users in India were men, the sample size of women in the survey was nearly seven times higher than men.

## Conclusions

Hot spots and vulnerable districts in the hot spots have to be given priority for targeted intervention. Health awareness programs, especially for ST women have to be implemented. Alcohol policy of individual states and UT appears to be the key determinant of alcohol use in India. Unfettered access to alcohol in most states has led to high prevalence. In a low middle-income country like India, the amount of money spent on tobacco and alcohol by millions of Indians on a daily basis would have a direct bearing on the quality of life. Strong anti-alcohol policies have to be implemented strictly across India to protect the financial, mental, and physical health of millions of Indian families.

## Data Availability Statement

Publicly available datasets were analyzed in this study. This data can be found at: https://www.dhsprogram.com.

## Ethics Statement

The studies involving human participants were reviewed and approved by the Institutional Review Board of International Institute for Population Sciences, Mumbai. Verbal, informed consent was obtained from respondents aged ≥18 years. The datasets of this study are available for public use at https://www.dhsprogram.com. Authorization to use the NFHS-4 datasets for this manuscript was obtained from demographic and health survey (DHS) program. Written informed consent for participation was not required for this study in accordance with the national legislation and the institutional requirements.

## Author Contributions

KB, WP, and PB contributed to the study design, data collection, data analysis, figures, data interpretation, literature search, and writing. RR, SC, and SB contributed to the literature search, data analysis, and interpretation. All authors contributed to the article and approved the submitted version.

## Conflict of Interest

The authors declare that the research was conducted in the absence of any commercial or financial relationships that could be construed as a potential conflict of interest.

## Publisher's Note

All claims expressed in this article are solely those of the authors and do not necessarily represent those of their affiliated organizations, or those of the publisher, the editors and the reviewers. Any product that may be evaluated in this article, or claim that may be made by its manufacturer, is not guaranteed or endorsed by the publisher.

## Abbreviations

SDI: Sociodemographic Indices
NFHS-4: National Family Health Survey-4
NE: North-East
ST: Scheduled Tribe
DHS: Demographic and Health Survey program.
