# Supplementary material for: Epidemiology, Hot Spots, and Sociodemographic Risk Factors of Alcohol Consumption in Indian Men and Women: Analysis of National Family Health Survey-4 (2015-16), a Nationally Representative Cross-Sectional Study
Source: Front Public Health. 2021 Aug 27;9:617311. doi: 10.3389/fpubh.2021.617311 (PMC8429933; doi:10.3389/fpubh.2021.617311)
Supplement: Supplementary file 1 [file Table_1.DOCX]

**Supplementary Table 1: Prevalence of alcohol consumption in hot spot districts of India**

| State | Popula-tion density (per sq.km) | Average alcohol consumption (%) | Alcohol hot spot districts |
| --- | --- | --- | --- |
| Andaman & Nicobar | 46 | Men: 51.7  Women: 2.5 | Nicobar |
| Arunanchal Pradesh | 17 | Men: 59.0  Women: 26.3 | Anjaw, Changlang, East Kameng, East Siang, Lohit, Lower Dibang Valley, Papum Pare, Tirap, Upper Subansiri , West Kameng, Upper Siang, Dibang Valley, West Siang, Tawang, Lower Subansiri |
| Assam | 397 | Men: 35.6  Women: 6.9 | Chirang , Dhemaji, Dibrugarh, Jorhat, Karbi Anglong, Lakhimpur, Marigaon, Dima Hasao, Sivasagar , Sonitpur, Tinsukia, Udalguri |
| Chhattisgarh | 189 | Men: 52.7  Women: 5.0 | Bastar, Bilaspur, Dhamtari, Durg , Jashpur, Uttar Bastar Kanker, Koriya , Narayanpur, Raigarh, Raipur, Rajnandgaon, Surguja, Dakshin Bastar Dantewada, Bijapur, Korba, Janjgir-Champa |
| Jharkhand | 414 | Men: 39.3  Women: 4.1 | Pashchimi Singhbhum, Lohardaga, Simdega, Gumla, Khunti, Ranchi, Saraikela-kharsawan |
| Kerala | 859 | Men: 37.0  Women: 1.6 | Pathanamthitta, Ernakulam, Alappuzha |
| Manipur | 122 | Men: 52.2  Women: 6.1 | Bishnupur, Chandel, Churachandpur, Imphal East, Imphal West, Senapati, Tamenglong, Ukhrul |
| Meghalaya | 132 | Men: 44.6  Women: 2.1 | East Garo Hills, Jaintia Hills, Ri Bhoi, South Garo Hills, West Garo Hills |
| Mizoram | 52 | Men: 49.6  Women: 5.0 | Aizawl, Champhai, Kolasib, Lunglei, Mamit, Serchhip |
| Nagaland | 119 | Men: 39.0  Women: 3.3 | Dimapur , Kiphire, Mokokchung, Mon, Peren, Phek, Wokha |
| Odisha | 269 | Men: 39.3  Women: 2.4 | Debagarh, Jharsuguda, Kendujhar, Koraput, Mayurbhanj, Nabarangapur, Nuapada, Subarnapur, Sundargarh, Malkangiri, Dhenkanal, Kandhamal, Anugul, Sambalpur |
| Sikkim | 86 | Men: 51.2  Women: 23.0 | North, West, South, East |
| Tamil Nadu | 555 | Men: 46.7  Women: 0.4 | Sivaganga, Chennai, Cuddalore, Thanjavur, Namakkal, Thiruvallur, Thiruvarur, Coimbatore, Tiruppur, Nagappattinam, Dindigul |
| Telangana | 312 | Men: 53.9  Women: 8.8 | Karimnagar, Medak, Nalgonda, Warangal, Khammam |
| Tripura | 350 | Men: 57.6  Women: 4.8 | Dhalai, North Tripura, South Tripura, West Tripura |
