# Supplementary material for: Epidemiology, Hot Spots, and Sociodemographic Risk Factors of Alcohol Consumption in Indian Men and Women: Analysis of National Family Health Survey-4 (2015-16), a Nationally Representative Cross-Sectional Study
Source: Front Public Health. 2021 Aug 27;9:617311. doi: 10.3389/fpubh.2021.617311 (PMC8429933; doi:10.3389/fpubh.2021.617311)
Supplement: Supplementary file 3 [file Table_3.DOCX]

STROBE Statement—Checklist of items that should be included in reports of ***cross-sectional studies***

|  | Item No | Recommendation |
| --- | --- | --- |
| **Title and abstract** | 1 | (*a*) Indicate the study’s design with a commonly used term in the title or the abstract  Study design (cross sectional study) is given in the title |
|  |  | (*b*) Provide in the abstract an informative and balanced summary of what was done and what was found  Abstract, Paragraphs 2, 3 and 4 |
| Introduction | | |
| Background/rationale | 2 | Explain the scientific background and rationale for the investigation being reported  Introduction, Paragraphs 1 and 2 |
| Objectives | 3 | State specific objectives, including any prespecified hypotheses  Introduction, Paragraph 2 |
| Methods: This is a secondary data analysis of the nationally representative cross-sectional study conducted by Indian Institute of Population Science, Mumbai, and was funded by USAID and UNICEF. The datasets were obtained from the Demographic Health Survey program. | | |
| Study design | 4 | Present key elements of study design early in the paper  Methods, Paragraph 1 (Data source) |
| Setting | 5 | Describe the setting, locations, and relevant dates, including periods of recruitment, exposure, follow-up, and data collection  Methods, Paragraph 1 (Data source) |
| Participants | 6 | (*a*) Give the eligibility criteria, and the sources and methods of selection of participants  Methods, Paragraph 1 (Data source) |
| Variables | 7 | Clearly define all outcomes, exposures, predictors, potential confounders, and effect modifiers. Give diagnostic criteria, if applicable  Methods, Paragraph 2 (Statistical analysis) |
| Data sources/ measurement | 8 | For each variable of interest, give sources of data and details of methods of assessment (measurement). Describe comparability of assessment methods if there is more than one group  Methods, Paragraphs 2 and 3 (Statistical analysis and spatial analysis) |
| Bias | 9 | Describe any efforts to address potential sources of bias  Methods, Paragraph 1 (Data source) |
| Study size | 10 | Explain how the study size was arrived at  Methods, Paragraph 1 (Data source) |
| Quantitative variables | 11 | Explain how quantitative variables were handled in the analyses. If applicable, describe which groupings were chosen and why  N/A (All the variables are qualitative) |
| Statistical methods | 12 | (*a*) Describe all statistical methods, including those used to control for confounding  Methods, Paragraph 2 (Statistical analysis) |
|  |  | (*b*) Describe any methods used to examine subgroups and interactions  N/A |
|  |  | (*c*) Explain how missing data were addressed  Methods, Paragraph 2 (Statistical analysis) |
|  |  | (*d*) If applicable, describe analytical methods taking account of sampling strategy  Methods, Paragraph 1 |
|  |  | (*e*) Describe any sensitivity analyses  N/A |
| Results | | |
| Participants | 13 | (a) Report numbers of individuals at each stage of study—eg numbers potentially eligible, examined for eligibility, confirmed eligible, included in the study, completing follow-up, and analysed  Table 1, Supplementary table 2 |
|  |  | (b) Give reasons for non-participation at each stage  N/A |
|  |  | (c) Consider use of a flow diagram  N/A |
| Descriptive data | 14 | (a) Give characteristics of study participants (eg demographic, clinical, social) and information on exposures and potential confounders  Supplementary table 2, Table 1 |
|  |  | (b) Indicate number of participants with missing data for each variable of interest  N/A |
| Outcome data | 15 | Report numbers of outcome events or summary measures  Table 1, Figures 1 and 2 |
| Main results | 16 | (*a*) Give unadjusted estimates and, if applicable, confounder-adjusted estimates and their precision (eg, 95% confidence interval). Make clear which confounders were adjusted for and why they were included  Table 1, Results, Paragraphs 7-10 |
|  |  | (*b*) Report category boundaries when continuous variables were categorized  N/A |
|  |  | (*c*) If relevant, consider translating estimates of relative risk into absolute risk for a meaningful time period  N/A |
| Other analyses | 17 | Report other analyses done—eg analyses of subgroups and interactions, and sensitivity analyses  N/A |
| Discussion | | |
| Key results | 18 | Summarise key results with reference to study objectives  Discussion, Paragraphs 2, 3, 4 and 5 |
| Limitations | 19 | Discuss limitations of the study, taking into account sources of potential bias or imprecision. Discuss both direction and magnitude of any potential bias  Discussion, Paragraph 6 |
| Interpretation | 20 | Give a cautious overall interpretation of results considering objectives, limitations, multiplicity of analyses, results from similar studies, and other relevant evidence  Discussion, Paragraphs 1, 2, 3, 4 and 5 |
| Generalisability | 21 | Discuss the generalisability (external validity) of the study results  Conclusion |
| Other information | | |
| Funding | 22 | Give the source of funding and the role of the funders for the present study and, if applicable, for the original study on which the present article is based  Methods, Paragraph 1 |
